# Applications and challenges of multi-omics approaches in lung cancer research and precision treatment

**DOI:** 10.3389/fgene.2025.1722368

**Published:** 2026-03-23

**Authors:** Shouyu Zhang, Renjie Xu, Qian Zheng, Bojiang Chen, Weimin Li

**Affiliations:** 1 Department of Pulmonary and Critical Care Medicine, West China Hospital, State Key Laboratory of Respiratory Health and Multimorbidity, Sichuan University, Chengdu, Sichuan, China; 2 Institute of Respiratory Health, Frontiers Science Center for Disease-related Molecular Network, West China Hospital, Sichuan University, Chengdu, Sichuan, China; 3 Precision Medicine Center, Precision Medicine Key Laboratory of Sichuan Province, West China Hospital, Sichuan University, Chengdu, Sichuan, China; 4 The Research Units of West China, Chinese Academy of Medical Sciences, West China Hospital, Chengdu, Sichuan, China; 5 Institute of Respiratory Health and Multimorbidity, West China Hospital, Sichuan University, Chengdu, Sichuan, China

**Keywords:** applications, challenges, lung cancer, multi-omics, precision medicine

## Abstract

Lung cancer is one of the most common cancers worldwide and one of the leading causes of cancer death, with a heavy disease burden and severe public health challenges. Multi-omics techniques, such as genomics, proteomics, metabolomics, and radiomics, play a crucial role in the early diagnosis and treatment of lung cancer, revealing the molecular characteristics and mechanisms of lung cancer, and have significant clinical application value. However, it also faces numerous challenges, such as data issues, “black box” problems, and ethical and legal concerns. How to leverage strengths while mitigating weaknesses, achieve clinical translation of technology, and serve patients more effectively deserves our deep reflection. This article reviews the specific applications and challenges of multi-omics methods in lung cancer research and personalized treatment.

## 1 Introduction

Lung cancer is one of the most common cancers in the world and one of the main causes of cancer death. According to GLOBOCAN 2020 statistics (Sung et al., 2021; Li C. et al., 2023), there are about 2.2 million new cases of lung cancer worldwide every year, accounting for 11.4% of all new cancers. The number of deaths caused by lung cancer is about 1.8 million, accounting for 18% of all cancer deaths. Consequently, lung cancer carries a substantial disease load (Bray et al., 2024; Zhou et al., 2024), leading to highly critical public health issues. There’s an immediate need to hasten comprehensive research and accurate lung cancer therapy, aiming to lower lung cancer deaths and enhance patient quality of life.

**FIGURE 1:**
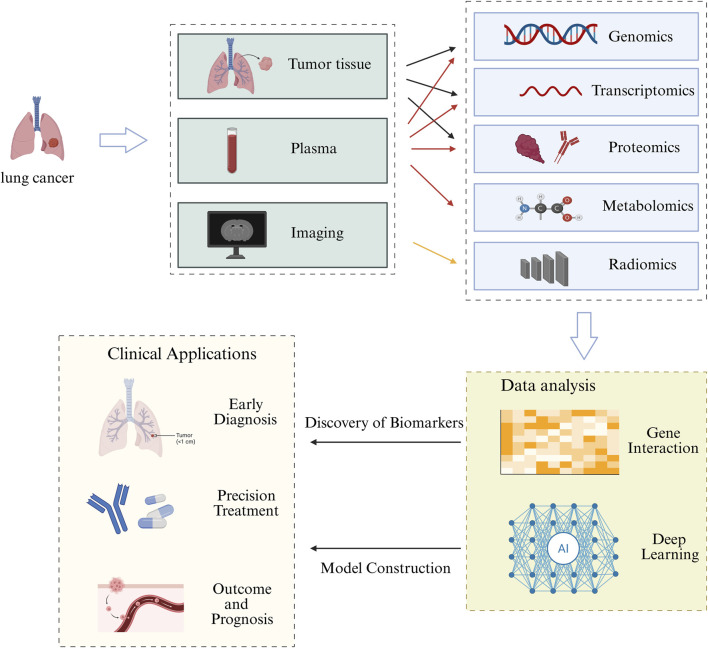
The Processes and Applications of Multi-omics Approaches. This figure consists of four components: (1) We can obtain clinical information, such as tumor tissue, blood samples, and imaging data from lung cancer patients; (2) Different sample types correspond to different omics analysis methods. Genomics, transcriptomics, and proteomics can be used to analyze tumor tissues. Genomics, transcriptomics, proteomics, and metabolomics can be used to analyze plasma, and radiomics can be used to analyze image data; (3) Discovery of biomarkers and construction of models through analysis of multiple-omics data; (4) Clinical application of multi-omics approaches.

Faced with such a significant challenge, the evolution of multi-omics technology has been progressing steadily. Multi-omics technology studies the characteristics, changes, and life activity patterns of organisms at a holistic level. It overcomes the limitations of single-omics approaches by uncovering information contained in multi-dimensional data, revealing regulatory relationships between molecules or cells, as well as the intrinsic laws and operational mechanisms of living organisms (He X. et al., 2023). It includes genomics, transcriptomics, proteomics, metabolomics, radiomics, and other technologies, which can reveal the molecular characteristics and related mechanisms of tumors from many aspects and different dimensions. It can help people better understand the path of tumor occurrence, development, and metastasis, and provide a new perspective for understanding tumors. For example, Zhang et al. elucidated the prognostic significance of mitochondrial-related genes in lung adenocarcinoma through transcriptomic and single-cell RNA sequencing data. They classified lung adenocarcinoma into distinct molecular subtypes, developed an AI-based prognostic model, and validated its accuracy across multiple datasets. (Zhang et al., 2024). Li et al. integrated genomics, transcriptomics, proteomics, and pathology to uncover the mechanisms behind poor prognosis in solid-predominant adenocarcinoma (linked to reduced TTF-1 and Napsin-A expression levels and increased microenvironmental resistance), while also highlighting the potential of immunotherapy in this subtype. (Li F. et al., 2023). Li et al. identified PSMD12, a key gene associated with brain metastasis in lung adenocarcinoma, through tumor mRNA expression profiling, which was further validated using single-cell RNA sequencing data and spatial transcriptomics. (Li et al., 2025). These findings collectively demonstrate the widespread use of multi-omics approaches in scientific research. By integrating multi-omics technology, we are able to precisely diagnose lung cancer early and develop intervention strategies, gain a profound insight into lung cancer’s drug resistance, and lay a robust theoretical and practical foundation for targeted medical practices. This article reviews the specific applications and challenges of multi-omics methods in lung cancer research and personalized treatment. (Figure 1).

## 2 Genomics

The definition of genomics was proposed by Thomas H. Roderick, an American geneticist, in 1986. It is a science that uses a variety of methods, integrates multiple disciplines, and analyzes, explains, compares, and functionally identifies the genomic information of organisms through measurement. It can be divided into functional genomics, structural genomics, epigenomics, population genomics, metagenomics, etc. (Gordon, 2002; Kadakkuzha and Puthanveettil, 2013) With the emergence of genomics, the next-generation sequencing technology (NGS) has gradually replaced the traditional sequencing methods (Goodwin et al., 2016) due to its characteristics of high speed, low cost, high throughput, high sensitivity, and so on, making it more crucial in cancer research and targeted medical practices.

The genomic alterations of lung cancer with feasible targeted therapy discovered by genomics make lung cancer management have broad prospects. Among them, driver gene mutations have the greatest clinical significance as they can promote the proliferation of tumor cells and complete the process of invasion and metastasis (Dan et al., 2024), including EGFR, KRAS, ALK, erbB2, BRAF, and other genes (Liu et al., 2017). Concurrently, genes that suppress tumors, including TP53, STK11, ATM, and APC, among others, also play a key role (Greulich, 2010). Knudson hypothesis (Valladares Ayerbes et al., 2008) believes that it requires the inactivation of both alleles to cause cancer. In addition, genomics also found some important copy number variations: the most common alteration is the increase of chromosome 5p (Witt, 2011). Chanida and colleagues (Vinayanuwattikun et al., 2016) identified two new focal alterations, namely, chromosome 12q14.1 containing the proto-oncogene agap2 and chromosome 16p12.1 containing the potential proto-oncogene RBBP6. Alterations in gene dosage could lead to the occurrence and development of tumors.

At present, there are still some potential therapeutic targets for non-small cell lung cancer (NSCLC). Nectin-4 is a cell adhesion molecule involved in tumor adhesion, proliferation, and metastasis (Nikanjam et al., 2025). It can be expressed on the cell membrane or cytoplasm, yet its expression is minimal in healthy tissues (Li K. et al., 2024). Nectin-4 overexpression exists in about 60% of NSCLC (Challita-Eid et al., 2016), and its amplification is observed in roughly 5%–10% of lung and breast cancer cases (Klümper et al., 2024). Enfortumab vedotin is a targeted drug currently used for Nectin-4 mutation treatment. In a phase II clinical trial (Muro et al., 2025), 6 of 43 non-adenocarcinoma patients finally achieved partial remission, but the results did not meet the expected anti-tumor activity. There is an immediate need for more comprehensive research. The folate transporter protein, Folate Receptor α (FRα), promotes tumor cell metabolism by providing folate and enhances folate uptake in cancer tissues (Liu et al., 2025). It has a strong ability to distinguish the histological types of lung cancer, showing a 74% positive rate in adenocarcinoma and 13% in squamous cell carcinoma (Tamura et al., 2020). Researches indicate that among lung adenocarcinoma patients undergoing surgical treatment, high expression of FRα is associated with improved overall survival (O'Shannessy et al., 2012). Some studies have also shown that FRα is associated with the pathways of some tumors. Hansen et al. (2015) found that folic acid activated the proto-oncogene STAT3 through FRα in a JAK-dependent manner, in which gp130 was the transduction receptor of this pathway.

Currently, tissue biopsy remains the primary method for diagnosing lung cancer and helps in classifying tumor subtypes, which guides treatment decisions and prognosis for cancer patients. The available chemotherapy and targeted drugs have significantly boosted survival rates for lung cancer patients. Because of the disadvantages of tissue biopsy, such as invasiveness, high cost, and long reporting time, liquid biopsy came into being. Due to its small trauma, convenient access to materials, and good patient compliance, it is receiving more and more research. Notably, tumor samples obtained through tissue biopsy can only represent a part of the lesion and do not fully reflect tumor heterogeneity and metastatic potential. This method gathers information about tumors by detecting the patient’s body fluids, mainly detecting circulating tumor DNA (ctDNA), circulating tumor cells, and exosomes (Pantel and Speicher, 2016; Ren et al., 2024). The body fluid includes blood, saliva, sputum, urine, pleural effusion, cerebrospinal fluid, bronchoalveolar lavage fluid, etc., but blood is the most commonly used body fluid (Li et al., 2022; Li L. et al., 2024). The initial application of liquid biopsy involved genotyping individuals with EGFR mutation positivity and advanced NSCLC (Kwapisz, 2017). Wu et al. (2015) found a 98.2% specificity in identifying EGFR mutations through ctDNA, with a sensitivity rate of 76.7%. As technology advances, the use of liquid biopsy has diversified increasingly. A study on ctDNA-MRD found that (John et al., 2024), the median time of MRD event positivity in patients in osimertinib and placebo groups was 4.7 months earlier than that of DFS event at the follow-up of 3 years, indicating that ctDNA has a certain ability to evaluate whether tumor recurrence. Another trial also showed that (Bazan Russo et al., 2025), the better prognosis of osimertinib alone and osimertinib combined with chemotherapy was related to the early clearance of plasma EGFR, which also indicated the prognostic role of ctDNA. In addition, the detection of methylation features on ctDNA was used for early screening of lung cancer. He J. et al. (2023) employed image features and ctDNA methylation features to establish a model to predict the benign and malignant lung nodules, with an AUC of up to 0.91. Wang Z. et al. (2023) detected methylation sites on ctDNA by PCR techniques, and the detection rate of lung cancer reached 90.6%.

Genomics is relatively mature at present, with rich application scenarios and targeted drugs for different sites are widely used in clinical practice. It should be noted that, in addition to the targets we are familiar with, the targeted drug effect of emerging targets does not seem to be ideal. Whether more targeted drugs can be developed to prolong the survival period of patients and improve the quality of life is a challenge currently. Furthermore, for liquid biopsy, the low level of plasma ctDNA, the DNA noise of non-tumor cells, and the influence of cell aging will limit this non-invasive diagnosis and treatment technology. How to balance the advantages and disadvantages is a problem we should think about.

## 3 Proteomics

Australian scientist Marc Wilkins introduced the concept of proteome in 1994 (Tyers and Mann, 2003; Manfredi et al., 2019), encompassing all proteins an organism produces. Proteomics is the science that studies the interaction and change rules of all proteins in the organism (Abbasian et al., 2022). Proteomic detection techniques such as immunoblotting and gel electrophoresis are employed, but they have low flux and high complexity. With the development of technology, mass spectrometry (MS) has become the main detection method now. MS purified the protein from the organism using diverse methods first, followed by its separation via capillary electrophoresis or liquid chromatography. Charged ions will be produced by the separated protein in the chromatograph. The mass-to-charge ratio of these ions is measured to create a mass spectrum, so as to match with various proteins. This step is also the core principle of MS (Messner et al., 2023).

Proteomics is capable of exploring the protein changes related to the occurrence and evolution of lung cancer. These markers have great potential in the early diagnosis and treatment, therapeutic effect, and prognosis evaluation of lung cancer. In terms of diagnosis, a study on early NSCLC lung adenocarcinoma patients in the Philippines (Dimayacyac-Esleta et al., 2025) discovered 6240 differentially expressed quantitative proteins and finally identified 33 proteins with potential as biomarkers, providing new ideas for drug development. Chen et al. (2022) also showed that the overall survival period of patients with GAPDH, RAC1, ATCR2, and other protein-positive lung adenocarcinoma was remarkably long compared to those with negative expression. These proteins with significant changes in plasma can be used as new markers for early screening of lung cancer. In terms of therapeutic effect, Böttger et al. (2019) determined three markers, UGGT1, COL6A1, and MAP4, to predict the treatment response of NSCLC patients to cisplatin; Suwinski et al. (2019) and others found that osteopontin significantly affected the overall survival of squamous cell carcinoma patients receiving radiotherapy and chemotherapy, while erythropoietin and vascular endothelial growth factor had a greater impact on the overall survival of adenocarcinoma patients. In terms of classification and prognosis, Huo et al. (2024) used proteomics techniques to analyze paraffin samples of small cell lung cancer patients post-surgery, and classified them into three types based on different clinical outcomes and treatment responses. Findings indicated that proteomic subtype was an independent prognostic factor, which performed better than TNM staging and could guide the direction of precise treatment. Song et al. (2024) reported five lung cancer subtypes through the analysis of the South Korean NSCLC cohort. Subtype 4 is characterized by activation of the PI3K-Akt signal pathway, which is related to poor prognosis and high-frequency metastasis, but not to histological type. TIMP1 is a tumor-secreted protein. Research has found that (Dantas et al., 2023) its high expression is closely related to the poor prognosis of lung cancer patients. Lu and colleagues (Lu et al., 2024) established a model through five markers, ADAM10, MIF, TEK, THBS2, and MAOA, to predict the relapse-free survival period and overall survival period in stage I lung adenocarcinoma patients. Patients with elevated scores have a poor prognosis (with a risk ratio of 8.28).

Glycoproteomics is an important branch of proteomics. Glycosylation is the most common way of post-translational modification of proteins. Glycosylation levels are indicative of the body’s physiological and pathological functions, disease progression, and prognosis, and have a profound impact on the pathological spectrum of cancer (Lin et al., 2020; Lin and Lubman, 2024). The study on patients in the Philippines (Alvarez et al., 2023) showed a notable rise in the relative abundance of mannose and sialofucosylated N-glycans in tumor tissue, which was significantly increased, indicating the role of abnormal glycosylation in lung cancer. Zhang et al. (2025) discovered that glycoprotein CDCP1 can be selectively loaded into extracellular vesicles, which may be a key step in lung cancer metastasis and a target for anti-metastasis research. Additional research has indicated that (Zhao et al., 2024) brain metastasis of lung adenocarcinoma can be divided into two subtypes according to glycosylation status, and N-glycosylation plays an important role in tumor progression. Besides the exploration of markers, Zeng et al. (2021) found that N-glycosylation reduced the resistance of cells to cisplatin, thereby reducing the ability of cell invasion and metastasis, providing new ideas for cisplatin resistance. Similarly, Alvarez et al. (2022) found a glycoprotein inhibitor, pictilisib. The determination of lung cancer cells treated with pictilisib showed that the protein expression involved in cell apoptosis was upregulated, while the protein involved in mRNA processing and protein translation was downregulated, suggesting that pictilisib may be a potential drug for lung cancer treatment.

The above research shows that proteomics is of great significance and has broad prospects in cancer research and precision treatment. However, there are still several problems. Currently, the majority of research projects are characterized by a limited number of participants and a single patient group, so the universality of the research findings remains a topic for debate. In terms of glycoproteomics, current research focuses on N-glycosylation, with little research on O-glycosylation due to the lack of an enzyme capable of decomposing all O-glycans (Wilkinson and Saldova, 2020), butO-glycosylation also plays an important role in protein stability, signal transduction, immune response, etc. (Chia et al., 2016). In the field of lung cancer, the discovery of O-glycans is mainly due to the low expression level of glycosyltransferase in cancer cells, and truncated O-GalNAc polysaccharides containing O-GalNAc (Tn antigen) or sialylated O-GalNAc (sTn antigen) are more common. (Doud and Yeh, 2023). Tn antigen mediates tumor metastasis, while sTn antigen acts on tumor development. (Zhang et al., 2022). The above questions indicate a need for more in-depth investigation into proteomics to ensure its significant clinical application.

## 4 Metabolomics

Metabolomics is a science developed after genomics and proteomics. It mainly studies small molecular substances such as sugars, lipids, amino acids, nucleotides, etc., that have a molecular weight below 1000 daltons. The physiological or pathological process of an organism at a certain stage can be described by analyzing the difference or enrichment of these metabolites (Clish, 2015; He B. et al., 2022). Metabolomics research methods include nuclear magnetic resonance, liquid chromatography, and gas chromatography tandem mass spectrometry. The nuclear magnetic resonance technology has a limited detection scope and suffers from low resolution and sensitivity, but the preprocessing of the technology is simple and nondestructive. Mass spectrometry is difficult to identify new substances, nor is it suitable for the detection of thermally unstable and volatile substances. However, it stands as a commonly employed technique for detection, renowned for its exceptional sensitivity, detailed resolution, and high throughput. (Beckonert et al., 2007; Telu et al., 2016; Ovbude et al., 2024).

The energy metabolism of tumor cells is relatively special. When oxygen levels are adequate, tumor cells will still use glycolysis to generate energy, that is, aerobic glycolysis, also known as the Warburg effect (Vander Heiden et al., 2009). During glycolysis and the circulation of tricarboxylic acid, a rise in pyruvate levels suppresses the body’s immune reaction and promotes tumor cell growth (Hensley and DeBerardinis, 2015). The transformation of pyruvate into lactic acid will increase the internal environment’s acidity, thereby suppressing the immune reaction and facilitating tumor penetration. So it is also a mechanism for the occurrence and development of lung cancer (Choi et al., 2013). The glucose metabolism pathway of lung cancer is significantly abnormal (Liang et al., 2024). The significant increase of pyruvate dehydrogenase kinase can promote the Warburg effect in tumor cells, presenting a potential therapeutic target (Zhang et al., 2018). However, some studies have shown that tumor cells have the ability to choose different ways to generate energy according to environmental changes. Under hypoxic conditions, the glycolysis mode is dominant, while when the oxygen content is sufficient, the mode of oxidative phosphorylation is significantly upregulated (Gonzalez et al., 2023; Zhou et al., 2023). This finding indicates that in the treatment of lung cancer, it may be necessary to combine multiple metabolic pathways for targeted treatment (Cai H. et al., 2025).

Lipid metabolism is another important pathway in the evolution of lung cancer. Godzien et al. (2023) revealed an increase in lysophosphatidic acid levels in lung squamous cell carcinoma sufferers, alongside a rise in oxidized phosphatidylcholine levels in those with adenocarcinoma. Oxidized phosphatidylcholine is related to the mitosis of vascular endothelial cells and can promote tumor angiogenesis, hinting at a prospective therapeutic path. Wang et al. (2025) selected six lipids building models to predict early-onset lung cancer, with an AUC of 0.874, which is a new breakthrough in lung cancer screening. Another study (Guan et al., 2023) included 370 patients with benign nodules and 478 patients with lung cancer, and screened for carnitine and a variety of amino acid markers. Carnitine can transport fatty acids from the cytoplasm to mitochondria to form acylcarnitine, which can reflect the development of lung cancer. Jiang et al. (2024) determined the efficacy (disease control or disease progression) of six lipids in predicting chemo-immunotherapeutic response in patients with advanced NSCLC. The AUC of the model established by combining clinical factors is 0.87, providing a framework for the precise treatment of lung cancer.

The disorder of amino acid metabolism is a prominent feature of lung cancer. Histidine has an anti-inflammatory effect, and it can be transformed into histamine under the inflammatory effect of the tumor microenvironment. Histamine participates in the proliferation, metastasis, and stimulation of the immune response of tumor cells (Medina and Rivera, 2010). Histidine is also involved in nucleotide synthesis, offering a genetic foundation for tumor cell proliferation (Tong et al., 2009). Serine and glycine are two nonessential amino acids. Tumor cells have the ability to enhance the activity of phosphoglycerate dehydrogenase, which is involved in the synthesis of serine and glycine, relating to tumor growth and poor clinical outcomes of patients (Zhu et al., 2016). These two amino acids can also participate in the synthesis of glutathione, which can maintain the redox homeostasis in cells. So tumor cells can survive against oxidative stress when the metabolic rate of serine and glycine increases (He L. et al., 2022). Betaine, a kind of trimethylglycine, is a metabolite of choline and a methyl donor. It can inhibit the activation of inflammatory bodies, reduce endoplasmic reticulum stress, and tumor progression (Zhao et al., 2018). In the study of Shang et al. (2024), eight metabolites were selected to distinguish healthy people, patients with lung adenocarcinoma, and patients with lung squamous carcinoma, including 3-phosphate serine, d-lysine, leucine, and others. The increase of these metabolites indicates the specificity of lung cancer subtypes and the special metabolic reprogramming mode in lung squamous carcinoma (Chen et al., 2025).

Metabolomics, as a new discipline developed in the post-genomic era, has helped us deepen our understanding of another dimension of the disease process. Compared with genomics and proteomics, it has many advantages, such as a small amount of data, easy detection, and simple analysis. Despite extensive research in metabonomics, the role of cholesterol metabolism in the progression of lung cancer is still unclear. Various research indicates significant differences in cholesterol levels among lung cancer patients, warranting contemplation on the causes of this variation and offering insights for upcoming metabolomics studies.

## 5 Radiomics

Radiomics can extract both quantitative and qualitative features from image data, especially those high-dimensional features that are difficult to capture with the naked eye, so as to assist clinicians in making clinical decisions (Kumar et al., 2012; Lambin et al., 2012). The basic process of radiomics mainly includes: first, image pre-processing, that is, image data cleaning, discretization, normalization, etc., to reduce background noise interference; Then image segmentation is carried out to outline ROI; The next step is to continue to extract radiomics features from ROI for screening and dimension reduction, and finally build and verify a prediction model (Zhou et al., 2020; Shur et al., 2021).

**FIGURE 2:**
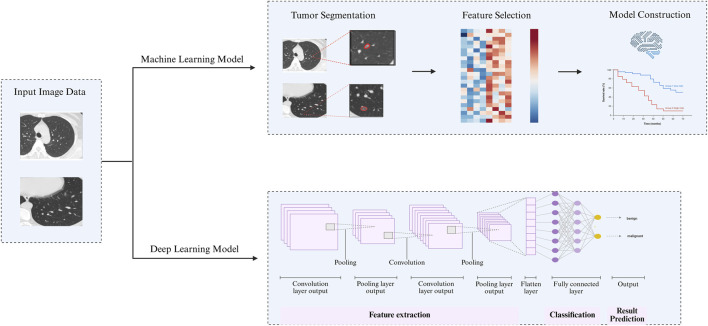
The Process of Radiomics. This figure consists of three components: (1) Input patient’s imaging data into the machine; (2) Machine Learning Model Method: segment the tumor site, delineate the ROI, select features, and finally construct a model; (3) Deep Learning Model Method: filter features through neural networks, classify and summarize features, and finally achieve result prediction.

The traditional radiomics mostly relies on manual delineation of the region of interest (ROI). Particularly in the past few years, advancements in artificial intelligence (AI) have enabled the integration of radiomics and deep learning, leading to the automation and enhanced intelligence of the entire process, now a primary focus in research (Zhang and Zhou, 2023). AI is a technology based on computer science that integrates multiple disciplines. It can mimic human responses and is a broad concept. In short, it allows machines to complete tasks that typically require human intelligence. Radiomics is a product of the intersection of medicine and engineering, using intelligent methods to solve clinical problems. (Pei et al., 2022). At present, machine learning and deep learning are mainly used for model construction. Machine learning is the process of using different algorithms to search for patterns from massive amounts of data, and to predict or describe new data by finding mapping relationships. Deep learning is a subset of machine learning, with its core being neural network structures. The length of these network structures is called the depth of the model, hence the name “deep learning”. Compared to machine learning, deep learning is better at processing “advanced features” such as images, speech, and text. (Choi et al., 2020) (Figure 2).

Radiomics is extensively employed in lung cancer diagnosis. In terms of the detection of pulmonary nodules and the prediction of benign and malignant nodules, Hu W. et al. (2024) trained the AI model through 506 CT images of pulmonary nodules and found that the accuracy of AI (the AUC is 0.88) exceeded that of clinicians (the AUC is 0.84 at most). Zhu and Fei (2025) employed the Cross-ViT network for feature extraction via two separate branches, achieving a 91% accuracy rate in classifying benign and malignant nodules on the LUNA16 dataset, superior to the majority of other classification techniques. Naseer et al. (2023) used a U-Net neural network to segment lung lobes and extract nodules, and the revised model achieved nearly 98% accuracy in nodule classification. In terms of pathological prediction of lung cancer, Boubnovski Martell et al. (2024) combined radiomics and metabolomics, and the final model achieved an F1 score of 0.78 in the identification of histological categories (normal tissue, adenocarcinoma, squamous cell carcinoma). Tyagi and Talbar (2023) used a multi-level 3D depth convolution neural network along with three classifiers for predicting TNM stages, and conducted asymmetric convolution under each stage label, achieving a total classification precision of 97%. Wang X. et al. (2023) combined genomics and radiomics to distinguish adenocarcinoma and squamous cell carcinoma, explaining the relationship between 26 gene mutation sites and these two subtypes, making the model interpretable. On the prediction of gene mutation, Lu et al. (2025) combined clinical data and image data to predict the expression of PD-L1 in NSCLC patients, and the AUC reached 0.85. Hashimoto et al. (2024) extracted 3951 radiomics information by analyzing the characteristics of tumor interior, tumor margin, and tumor periphery. In cases where a biopsy is unfeasible or the presence of a PD-L1 mutation remains undetermined, this model serves as an effective supplementary tool. Mahajan et al. (2024) enrolled 990 patients with lung adenocarcinoma in the preoperative image data. They extracted the radiomics characteristics and established a model to predict the EGFR mutation state, with an AUC of 0.88. Cao et al. (2025) used MRI data to predict EGFR mutations in NSCLC patients with brain metastases, and the AUC reached 0.93, which can provide direction for clinical decision-making.

Radiomics can also be used in the personalized treatment of lung cancer. In terms of lung nodule management, Landy et al. (2023) analyzed CT images of patients with suspected non-malignant nodules, enabling early screening and precise identification of low-risk nodules, along with the development of suitable follow-up strategies for them. In terms of efficacy prediction, Yang et al. (2025) combined clinical and pathological images with CT to predict the targeted therapeutic sensitivity of EGFR mutation in lung adenocarcinoma patients, and the AUC reached 0.84. Cai F. et al. (2025) predicted the treatment response of advanced NSCLC induction therapy by combining the extracted features of intratumoral and peritumoral regions with different algorithms, and the AUC was up to 0.93. Ventura and colleagues (Ventura et al., 2023) divided advanced NSCLC patients into two groups: one focusing on disease progression and the other on disease stability or remission in response to CKI-based chemotherapy and immunotherapy. The accuracy predicted by the median and skewness features was 0.75. In terms of prognosis prediction, Shimada et al. (2022) extracted 22 features to predict the probability of early recurrence in 0-IA stage NSCLC patients within 2 years after surgery, finding that features such as CT standard deviation, proportion of solid components, and bronchial translucency were associated with the patient’s relapse-free survival. The integrated learning model built by Gong et al. (2024) can predict the risk of brain metastasis in patients with advanced NSCLC, achieving an AUC of 0.91. The FAIS model established by Wang et al. (2022) can predict the efficacy of EGFR-TKI prognosis, achieving an AUC of 0.813, and it can identify patients with a high risk of TKI resistance.

Radiomics technology, especially the addition of AI, has brought a qualitative leap to the field of clinical medicine. It can assist doctors in making diagnosis, and even provide a treatment plan for reference, and consequently reduce the clinical burden of medical workers. In the future, AI is expected to develop into a big language model to assist clinical work more scientifically, intelligently, and accurately through human-computer interaction. However, at present, the number of models available for developing surgical strategies is limited, and there’s also a notable lack in predicting rare gene mutations. How to integrate multi-dimensional data of patients to develop a comprehensive prediction model may also be one of the research directions of precision medicine in the future.

## 6 Challenges faced by multi-omics approaches

**FIGURE 3:**
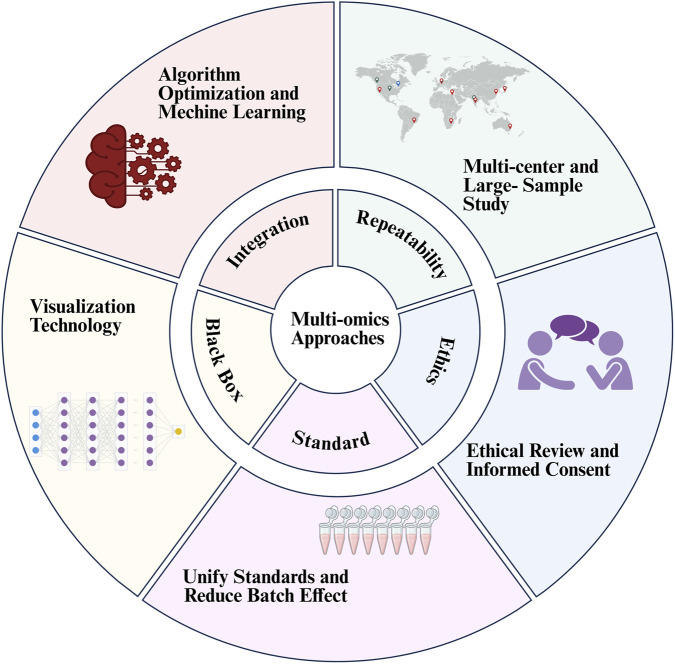
Challenges and Solutions Faced by Multi-omics Approaches. The inner circle points out the current challenges, and the outer circle provides corresponding solutions.

At present, the combined analysis of multi-omics methods plays an increasingly important role in cancer research and precision medicine, and has become the mainstream trend. However, multi-omics technology also faces many challenges. (Figure 3).

The first is data integration and repeatability. Data acquisition is the first challenge faced by the multi-omics method. Missing values are inevitable in this process. Contemplating the appropriate interpolation technique to adequately fill the data, minimize mistakes, and guarantee the precision of the ensuing analysis is valuable. Currently, the integration methods of multi-omics data include early, middle, and late integration. The early integration is to directly merge the data of different omics, also known as input layer or feature layer integration. This is the simplest integration method that can extract baseline features, but this will lead to the expansion of background noise and a latitude disaster. At the same time, data from different modalities must be aligned. The medium-term integration requires independent processing of each set of data before merging. It can retain the relationship between different omics data, with the interaction analysis being more prominent. The processing depth of each set of data can also be customized, and there are multiple architectures to choose from. But the complexity of the algorithm is relatively high, making it difficult to find the optimal depth for processing each set of data, which can lead to the loss of certain features. The late integration is completely opposite to the early integration. It analyzes the data of different omics first and then integrates the results. It can be trained in each modality, but it weakens the interaction of different omics (Baltrusaitis et al., 2019; Chakraborty et al., 2024; Gao and Tang, 2025; Mena et al., 2025). If omics data is missing, the entire omics system needs to have robustness to ensure that other modalities can still make predictions. We must confront the issue of determining the integration mode that minimally affects test outcomes. At present, there are various methods or platforms available for processing multi-omics data, such as the Bayesian method, fusion method, similarity-based method, etc., as well as platforms such as CGDV80 and SLIDE. However, there are significant differences in format between different methods or platforms, and there is also a lack of appropriate filtering criteria for data preprocessing. Therefore, we need to build a platform for joint analysis of multiple datasets. (Jha et al., 2017; Subramanian et al., 2020). Besides, most studies are single-center studies and have a small sample size. For example, in the multi-omics studies provided by TAO for non-invasive detection of gastrointestinal cancer, only 42 individuals had four types of omics data, and 84 individuals had three types of omics data (Tao et al., 2023); Hu et al. only included 100 patients with renal clear cell carcinoma for analysis, such as whole exome sequencing and whole transcriptome sequencing. (Hu J. et al., 2024). In addition, the different collection, transportation, storage, processing, and operating procedures of tumor tissue samples, as well as the different data integration and analysis methods, bring about the problem of data repeatability. Different scripts and analysis software, and many studies that do not describe parameter allocation and analysis methods, make many researches difficult to replicate, which is also a problem we need to solve.

Data standardization and clinical analysis are the second problem. Different omics methods differ in their measurement methods, units, and data formats. Genomics data includes gene sequence data, single-nucleotide variation, copy number variation, etc., while proteomics data is mainly protein expression level or post-translational modification data, and metabolomics is mainly presented in the concentration of metabolites, making standardization and normalization processing complex. Even if using the same measurement method, the data measured at different times, different places, and even different people may cause differences in results. Known as the batch effect, this phenomenon can escalate the occurrence of false positives or negatives, leading to significant errors in experiments. On the other hand, it is also a challenge to transform the experimental findings of multi-omics approaches into meaningful clinical conclusions. Multi-omics analysis explains a complete patho-physiological process. How to connect it with known markers and targets is still an unresolved problem (Sahu et al., 2025). Some experts also pointed out that tumor heterogeneity will lead to differences in multi-omics data, but multi-omics analysis can only find average signals, and the analysis of heterogeneity is not obvious (Feng et al., 2025).

The third is the “black box” problem. The “black box” problem of AI has been criticized for a long time. Failing to solve the “black box” issue will render AI unsafe and untrustworthy. The logic form of AI uses numbers as the carrier and runs through different algorithms. It is difficult to explain how it comes to a conclusion. In deep learning, there are a large number of hierarchical progressive neural networks. In the first layer of the network, the model only processes basic features such as size, color, and shape. In subsequent networks, the model recombines or extracts features from the previous layer to form more complex high-dimensional features that humans often cannot understand. (Aravazhi et al., 2025) .Therefore, the mathematical logic behind each operation of AI cannot be reproduced by human beings, and we will not know what AI is doing. To understand the decisions behind artificial intelligence, it is necessary to trace back to the trillions of parameter changes and cascading reactions inherent in the model, which is an unattainable process. For example, there may be deception tests in the model training. AI knows the answers that the inputters want to get, resulting in the expected output every time, but in the actual test, AI will give completely opposite answers. In clinical practice, explaining the principle of AI’s answer to patients has become an urgent problem to be solved.

Ethical and legal concerns are the fourth aspect. Multi-omics research will inevitably include a large amount of patient privacy data, and it's impossible for data to be entirely anonymous. Once data leakage occurs in the process of receipt, storage, analysis, and sharing, patients' privacy will be violated, posing a big challenge in data protection. Furthermore, multi-omics data may be repeatedly used for a long time, and patients' informed consent is complex. It is also a challenge to explain the use and risks of multi-omics technology to patients and other non-professionals in terms of communication. For example, according to reports, in 2019, the National Cerebral and Cardiovascular Center in Osaka, Japan discovered 158 cases of using patient data for research without going through the normal established procedures, of which 2 cases were also published in papers. In these cases, patients were not given the opportunity to refuse to participate in the study, which violated their right to informed consent; In 2015, the Philippines approved the use of dengue fever vaccine, but the vaccine company stated at the end of 2017 that the vaccine could cause serious illness in healthy individuals. This is a government regulatory loophole, where companies disguise clinical trials as public health programs, prompting us to strengthen project transparency and evaluation standards. Certainly, the wrong interpretation of the omics data may lead to missed diagnosis, misdiagnosis, or even improper treatment plans. Under this kind of medical accident, there is controversy about how to divide the specific responsibilities.

The fifth is the issue of economic costs. The price difference for different types of testing is substantial, with multi-gene testing costing around 2000-5000 yuan, while whole-exome or whole-genome sequencing costs between 18000-50000 yuan. The price of multi-omics combined testing is higher: the price of genome combined single-cell transcriptome sequencing is approximately 80,000 to 120,000 yuan per sample. The cost of single-cell ATAC-seq combined genome sequencing, suitable for epigenetic and genomic association analysis, can reach up to 100,000 to 150,000 yuan. Besides, the sample type also has a certain impact on the price. Blood sample processing is relatively simple, while tissue samples require dissociation, purification, and nucleic acid amplification, which increases costs by 30%–50%; FFPE samples require special preprocessing, and the cost per sample will also increase. Therefore, multi-omics methods can impose a heavy economic burden on patients. In addition, current medical insurance cannot cover all items, and the reimbursement ratios for different items are also different. Under such pressure, patients may choose low-priced but not comprehensive testing plans, or even give up such plans. This not only affects the quality of life of patients, but also reduces the practical application value of multi-omics methods.

## 7 Conclusion

At present, the field of multi-omics technology is extensively involved in diverse medical research areas and holds significant potential. AI’s advancement has infused it with fresh energy. The biomarkers and patho-physiological mechanisms discovered by the multi-omics technology have made great achievements in the early detection of pulmonary nodules, the early diagnosis of lung cancer, the development of treatment plans, the prediction of therapeutic effect, and prognosis. They can help doctors make diagnosis to a certain extent, timely observe the changes in patients' condition, and adjust the treatment plan, providing new methods, new insights, and new ideas for lung cancer management. However, the standardization, analysis, and integration of omics data, how to achieve clinical transformation, the “black box” problem of AI, and the ethical and legal issues of scientific research are all inevitable challenges. Currently, multi-omics technologies are increasingly used for dynamic disease monitoring, overcoming the delays of traditional methods and tracking molecular changes in tumor development, progression, and treatment response. For example, genome monitoring can detect early gene mutations (such as EGFR, ALK, etc.) in lung cancer to enable early diagnosis and intervention. During treatment, monitoring the expression levels of PD-L1 and tumor-specific metabolites helps assess disease progression. Additionally, targeting specific mutations (like T790M) guides therapy choices and aims to extend patient survival. In addition, single-cell multi-omics technology is becoming the current trend of development. It can obtain multidimensional information from the level of individual cells, breaking through the shortcomings of traditional techniques that are difficult to solve cell heterogeneity. It can identify rare cells, distinguish differences in different cell subpopulations, and avoid research biases that are one-size-fits-all. At the same time, it can shorten the target validation cycle by 3–6 months, accelerate basic scientific research and drug development processes, and promote efficient translation from experiments to clinical applications. It is believed that in the future, with the optimization and iteration of technology and continuous collaboration in multiple fields, multi-omics technology is expected to significantly reduce the mortality of lung cancer, improve the quality of life of patients, and reduce the burden of cancer.

## References

[B1] Abbasian M. H. Ardekani A. M. Sobhani N. Roudi R. (2022). The role of genomics and proteomics in lung cancer early detection and treatment. Cancers (Basel) 14 (20), 5144. 10.3390/cancers14205144 36291929 PMC9600051

[B2] Alvarez M. R. S. Zhou Q. Grijaldo S. J. B. Lebrilla C. B. Nacario R. C. Heralde F. M. 3rd (2022). An integrated mass spectrometry-based glycomics-driven glycoproteomics analytical platform to functionally characterize glycosylation inhibitors. Molecules 27 (12), 3834. 10.3390/molecules27123834 35744954 PMC9228227

[B3] Alvarez M. R. Zhou Q. Tena J. Barboza M. Wong M. Xie Y. (2023). Glycomic, glycoproteomic, and proteomic profiling of Philippine lung cancer and peritumoral tissues: case series study of patients stages I-III. Cancers (Basel) 15 (5), 1559. 10.3390/cancers15051559 36900350 PMC10001221

[B4] Aravazhi P. S. Gunasekaran P. Benjamin N. Z. Y. Thai A. Chandrasekar K. K. Kolanu N. D. (2025). The integration of artificial intelligence into clinical medicine: trends, challenges, and future directions. Dis. Mon. 71 (6), 101882. 10.1016/j.disamonth.2025.101882 40140300

[B5] Baltrusaitis T. Ahuja C. Morency L. P. (2019). Multimodal machine learning: a survey and taxonomy. IEEE Trans. Pattern Anal. Mach. Intell. 41 (2), 423–443. 10.1109/tpami.2018.2798607 29994351

[B6] Bazan Russo T. D. Pepe F. Gristina V. Gottardo A. Russo G. Scimone C. (2025). Recent advances in liquid biopsy for precision oncology: emerging biomarkers and clinical applications in lung cancer. Future Oncol. 21 (21), 2803–2821. 10.1080/14796694.2025.2542051 40762271 PMC12408062

[B7] Beckonert O. Keun H. C. Ebbels T. M. Bundy J. Holmes E. Lindon J. C. (2007). Metabolic profiling, metabolomic and metabonomic procedures for NMR spectroscopy of urine, plasma, serum and tissue extracts. Nat. Protoc. 2 (11), 2692–2703. 10.1038/nprot.2007.376 18007604

[B8] Böttger F. Schaaij-Visser T. B. de Reus I. Piersma S. R. Pham T. V. Nagel R. (2019). Proteome analysis of non-small cell lung cancer cell line secretomes and patient sputum reveals biofluid biomarker candidates for cisplatin response prediction. J. Proteomics 196, 106–119. 10.1016/j.jprot.2019.01.018 30710758

[B9] Boubnovski Martell M. Linton-Reid K. Hindocha S. Chen M. Moreno P. Álvarez-Benito M. (2024). Deep representation learning of tissue metabolome and computed tomography annotates NSCLC classification and prognosis. NPJ Precis. Oncol. 8 (1), 28. 10.1038/s41698-024-00502-3 38310164 PMC10838282

[B10] Bray F. Laversanne M. Sung H. Ferlay J. Siegel R. L. Soerjomataram I. (2024). Global cancer statistics 2022: GLOBOCAN estimates of incidence and mortality worldwide for 36 cancers in 185 countries. CA Cancer J. Clin. 74 (3), 229–263. 10.3322/caac.21834 38572751

[B11] Cai F. Guo Z. Wang G. Luo F. Yang Y. Lv M. (2025). Integration of intratumoral and peritumoral CT radiomic features with machine learning algorithms for predicting induction therapy response in locally advanced non-small cell lung cancer. BMC Cancer 25 (1), 461. 10.1186/s12885-025-13804-x 40082786 PMC11907900

[B12] Cai H. Zhang F. Xu F. Yang C. (2025). Metabolic reprogramming and therapeutic targeting in non-small cell lung cancer: emerging insights beyond the warburg effect. Front. Oncol. 15, 1564226. 10.3389/fonc.2025.1564226 40469185 PMC12133740

[B13] Cao P. Jia X. Wang X. Fan L. Chen Z. Zhao Y. (2025). Deep learning radiomics for the prediction of epidermal growth factor receptor mutation status based on MRI in brain metastasis from lung adenocarcinoma patients. BMC Cancer 25 (1), 443. 10.1186/s12885-025-13823-8 40075375 PMC11899356

[B14] Chakraborty S. Sharma G. Karmakar S. Banerjee S. (2024). Multi-OMICS approaches in cancer biology: new era in cancer therapy. Biochim. Biophys. Acta Mol. Basis Dis. 1870 (5), 167120. 10.1016/j.bbadis.2024.167120 38484941

[B15] Challita-Eid P. M. Satpayev D. Yang P. An Z. Morrison K. Shostak Y. (2016). Enfortumab vedotin antibody-drug conjugate targeting Nectin-4 is a highly potent therapeutic agent in multiple preclinical cancer models. Cancer Res. 76 (10), 3003–3013. 10.1158/0008-5472.Can-15-1313 27013195

[B16] Chen H. Lai X. Zhu Y. Huang H. Zeng L. Zhang L. (2022). Quantitative proteomics identified circulating biomarkers in lung adenocarcinoma diagnosis. Clin. Proteomics 19 (1), 44. 10.1186/s12014-022-09381-x 36404333 PMC9677906

[B17] Chen W. Xu Y. Liu H. (2025). Metabolomic characteristics and clinical implications in pathological subtypes of lung cancer. Cancer Screen. Prev. 4 (2), 98–106. 10.14218/csp.2025.00005

[B18] Chia J. Goh G. Bard F. (2016). Short O-GalNAc glycans: regulation and role in tumor development and clinical perspectives. Biochim. Biophys. Acta 1860 (8), 1623–1639. 10.1016/j.bbagen.2016.03.008 26968459

[B19] Choi S. Y. Collins C. C. Gout P. W. Wang Y. (2013). Cancer-generated lactic acid: a regulatory, immunosuppressive metabolite? J. Pathol. 230 (4), 350–355. 10.1002/path.4218 23729358 PMC3757307

[B20] Choi R. Y. Coyner A. S. Kalpathy-Cramer J. Chiang M. F. Campbell J. P. (2020). Introduction to machine learning, neural networks, and deep learning. Transl. Vis. Sci. Technol. 9 (2), 14. 10.1167/tvst.9.2.14 32704420 PMC7347027

[B21] Clish C. B. (2015). Metabolomics: an emerging but powerful tool for precision medicine. Cold Spring Harb. Mol. Case Stud. 1 (1), a000588. 10.1101/mcs.a000588 27148576 PMC4850886

[B22] Dan A. Burtavel L. M. Coman M. C. Focsa I. O. Duta-Ion S. Juganaru I. R. (2024). Genetic blueprints in lung cancer: foundations for targeted therapies. Cancers (Basel) 16 (23), 4048. 10.3390/cancers16234048 39682234 PMC11639944

[B23] Dantas E. Murthy A. Ahmed T. Ahmed M. Ramsamooj S. Hurd M. A. (2023). TIMP1 is an early biomarker for detection and prognosis of lung cancer. Clin. Transl. Med. 13 (10), e1391. 10.1002/ctm2.1391 37759102 PMC10533479

[B24] Dimayacyac-Esleta B. R. T. Mira F. D. Zarate L. M. Porras B. J. O. Juntilla D. L. A. Suñga L. B. L. (2025). Discovery of key candidate protein biomarkers in early-stage nonsmall cell lung carcinoma through quantitative proteomics. J. Proteome Res. 24 (4), 1701–1714. 10.1021/acs.jproteome.4c00764 40014793

[B25] Doud E. H. Yeh E. S. (2023). Mass spectrometry-based glycoproteomic workflows for cancer biomarker discovery. Technol. Cancer Res. Treat. 22, 15330338221148811. 10.1177/15330338221148811 36740994 PMC9903044

[B26] Feng S. Yin X. Shen Y. (2025). Artificial intelligence-powered precision: unveiling the tumor microenvironment for a new frontier in personalized cancer therapy. Intell. Med. 5 (2), 95–98. 10.1016/j.imed.2025.02.001

[B27] Gao F. Tang Y. (2025). Multimodal deep learning for cephalometric landmark detection and treatment prediction. Sci. Rep. 15 (1), 25205. 10.1038/s41598-025-06229-w 40651957 PMC12255727

[B28] Godzien J. Lopez-Lopez A. Sieminska J. Jablonowski K. Pietrowska K. Kisluk J. (2023). Exploration of oxidized phosphocholine profile in non-small-cell lung cancer. Front. Mol. Biosci. 10, 1279645. 10.3389/fmolb.2023.1279645 38288337 PMC10824250

[B29] Gong J. Wang T. Wang Z. Chu X. Hu T. Li M. (2024). Enhancing brain metastasis prediction in non-small cell lung cancer: a deep learning-based segmentation and CT radiomics-based ensemble learning model. Cancer Imaging 24 (1), 1. 10.1186/s40644-023-00623-1 38167564 PMC10759676

[B30] Gonzalez M. A. Lu D. R. Yousefi M. Kroll A. Lo C. H. Briseño C. G. (2023). Phagocytosis increases an oxidative metabolic and immune suppressive signature in tumor macrophages. J. Exp. Med. 220 (6), e20221472. 10.1084/jem.20221472 36995340 PMC10067971

[B31] Goodwin S. McPherson J. D. McCombie W. R. (2016). Coming of age: ten years of next-generation sequencing technologies. Nat. Rev. Genet. 17 (6), 333–351. 10.1038/nrg.2016.49 27184599 PMC10373632

[B32] Gordon S. (2002). Genomics and world health. Report of the advisory committee on health research. Trans. R. Soc. Trop. Med. Hyg. 96(6) 669–669. 10.1016/S0035-9203(02)90347-0

[B33] Greulich H. (2010). The genomics of lung adenocarcinoma: opportunities for targeted therapies. Genes Cancer 1 (12), 1200–1210. 10.1177/1947601911407324 21779443 PMC3092285

[B34] Guan X. Du Y. Ma R. Teng N. Ou S. Zhao H. (2023). Construction of the XGBoost model for early lung cancer prediction based on metabolic indices. BMC Med. Inf. Decis. Mak. 23 (1), 107. 10.1186/s12911-023-02171-x 37312179 PMC10262551

[B35] Hansen M. F. Greibe E. Skovbjerg S. Rohde S. Kristensen A. C. Jensen T. R. (2015). Folic acid mediates activation of the pro-oncogene STAT3 via the folate receptor alpha. Cell Signal 27 (7), 1356–1368. 10.1016/j.cellsig.2015.03.020 25841994

[B36] Hashimoto K. Murakami Y. Omura K. Takahashi H. Suzuki R. Yoshioka Y. (2024). Prediction of tumor PD-L1 expression in resectable non-small cell lung cancer by machine learning models based on clinical and radiological features: performance comparison with preoperative biopsy. Clin. Lung Cancer 25 (1), e26–e34.e26. 10.1016/j.cllc.2023.08.010 37673781

[B37] He B. Huang Z. Huang C. Nice E. C. (2022). Clinical applications of plasma proteomics and peptidomics: towards precision medicine. Proteomics Clin. Appl. 16 (6), e2100097. 10.1002/prca.202100097 35490333

[B38] He L. Endress J. Cho S. Li Z. Zheng Y. Asara J. M. (2022). Suppression of nuclear GSK3 signaling promotes serine/one-carbon metabolism and confers metabolic vulnerability in lung cancer cells. Sci. Adv. 8 (20), eabm8786. 10.1126/sciadv.abm8786 35594343 PMC9122323

[B39] He J. Wang B. Tao J. Liu Q. Peng M. Xiong S. (2023). Accurate classification of pulmonary nodules by a combined model of clinical, imaging, and cell-free DNA methylation biomarkers: a model development and external validation study. Lancet Digit. Health 5 (10), e647–e656. 10.1016/s2589-7500(23)00125-5 37567793

[B40] He X. Liu X. Zuo F. Shi H. Jing J. (2023). Artificial intelligence-based multi-omics analysis fuels cancer precision medicine. Seminars Cancer Biol. 88, 187–200. 10.1016/j.semcancer.2022.12.009 36596352

[B41] Hensley C. T. DeBerardinis R. J. (2015). *In vivo* analysis of lung cancer metabolism: nothing like the real thing. J. Clin. Invest 125 (2), 495–497. 10.1172/jci79188 25607834 PMC4319439

[B42] Hu J. Wang S. G. Hou Y. Chen Z. Liu L. Li R. (2024). Multi-omic profiling of clear cell renal cell carcinoma identifies metabolic reprogramming associated with disease progression. Nat. Genet. 56 (3), 442–457. 10.1038/s41588-024-01662-5 38361033 PMC10937392

[B43] Hu W. Zhang J. Zhou D. Xia S. Pu X. Cao J. (2024). A comparison study of artificial intelligence performance against physicians in benign–malignant classification of pulmonary nodules. Oncologie 26 (4), 581–586. 10.1515/oncologie-2023-0319

[B44] Huo Z. Duan Y. Zhan D. Xu X. Zheng N. Cai J. (2024). Proteomic stratification of prognosis and treatment options for small cell lung cancer. Genomics Proteomics Bioinforma. 22 (2), qzae033. 10.1093/gpbjnl/qzae033 38961535 PMC11423856

[B45] Jha V. Singh G. Kumar S. Sonawane A. Jere A. Anamika K. (2017). CGDV: a webtool for circular visualization of genomics and transcriptomics data. BMC Genomics 18 (1), 823. 10.1186/s12864-017-4169-5 29065857 PMC5655900

[B46] Jiang H. Li X. S. Yang Y. Qi R. X. (2024). Plasma lipidomics profiling in predicting the chemo-immunotherapy response in advanced non-small cell lung cancer. Front. Oncol. 14, 1348164. 10.3389/fonc.2024.1348164 39040440 PMC11260645

[B47] John T. Grohe C. Goldman J. W. Kato T. Laktionov K. K. Bonanno L. (2024). Molecular residual disease (MRD) analysis from the ADAURA trial of adjuvant (adj) osimertinib in patients (pts) with resected EGFR-Mutated (EGFRm) stage IB–IIIA non-small cell lung cancer (NSCLC). J. Clin. Oncol. 42 (16_Suppl. l), 8005. 10.1200/JCO.2024.42.16_suppl.8005

[B48] Kadakkuzha B. M. Puthanveettil S. V. (2013). Genomics and proteomics in solving brain complexity. Mol. Biosyst. 9 (7), 1807–1821. 10.1039/c3mb25391k 23615871 PMC6425491

[B49] Klümper N. Tran N. K. Zschäbitz S. Hahn O. Büttner T. Roghmann F. (2024). NECTIN4 amplification is frequent in solid tumors and predicts enfortumab vedotin response in metastatic urothelial cancer. J. Clin. Oncol. 42 (20), 2446–2455. 10.1200/jco.23.01983 38657187 PMC11227306

[B50] Kumar V. Gu Y. Basu S. Berglund A. Eschrich S. A. Schabath M. B. (2012). Radiomics: the process and the challenges. Magn. Reson Imaging 30 (9), 1234–1248. 10.1016/j.mri.2012.06.010 22898692 PMC3563280

[B51] Kwapisz D. (2017). The first liquid biopsy test approved. Is it a new era of mutation testing for non-small cell lung cancer? Ann. Transl. Med. 5 (3), 46. 10.21037/atm.2017.01.32 28251125 PMC5326656

[B52] Lambin P. Rios-Velazquez E. Leijenaar R. Carvalho S. van Stiphout R. G. Granton P. (2012). Radiomics: extracting more information from medical images using advanced feature analysis. Eur. J. Cancer 48 (4), 441–446. 10.1016/j.ejca.2011.11.036 22257792 PMC4533986

[B53] Landy R. Wang V. L. Baldwin D. R. Pinsky P. F. Cheung L. C. Castle P. E. (2023). Recalibration of a deep learning model for low-dose computed tomographic images to inform lung cancer screening intervals. JAMA Netw. Open 6 (3), e233273. 10.1001/jamanetworkopen.2023.3273 36929398 PMC10020880

[B54] Li W. Liu J. B. Hou L. K. Yu F. Zhang J. Wu W. (2022). Liquid biopsy in lung cancer: significance in diagnostics, prediction, and treatment monitoring. Mol. Cancer 21 (1), 25. 10.1186/s12943-022-01505-z 35057806 PMC8772097

[B55] Li C. Lei S. Ding L. Xu Y. Wu X. Wang H. (2023). Global burden and trends of lung cancer incidence and mortality. Chin. Med. J. Engl. 136 (13), 1583–1590. 10.1097/cm9.0000000000002529 37027426 PMC10325747

[B56] Li F. Wang S. Wang Y. Lv Z. Jin D. Yi H. (2023). Multi-omics analysis unravels the underlying mechanisms of poor prognosis and differential therapeutic responses of solid predominant lung adenocarcinoma. Front. Immunol. 14, 1101649. 10.3389/fimmu.2023.1101649 36845145 PMC9946976

[B57] Li K. Zhou Y. Zang M. Jin X. Li X. (2024). Therapeutic prospects of nectin-4 in cancer: applications and value. Front. Oncol. 14, 1354543. 10.3389/fonc.2024.1354543 38606099 PMC11007101

[B58] Li L. Jiang H. Zeng B. Wang X. Bao Y. Chen C. (2024). Liquid biopsy in lung cancer. Clin. Chim. Acta 554, 117757. 10.1016/j.cca.2023.117757 38184141

[B59] Li X. Zhang J. Zhang M. Jiang W. Jia D. Wang R. (2025). Integrating multi-omics to unveil PSMD12 as a critical gene in promoting brain metastases of lung adenocarcinoma. J. Transl. Med. 23 (1), 668. 10.1186/s12967-025-06680-3 40528170 PMC12175416

[B60] Liang S. Cao X. Wang Y. Leng P. Wen X. Xie G. (2024). Metabolomics analysis and diagnosis of lung cancer: insights from diverse sample types. Int. J. Med. Sci. 21 (2), 234–252. 10.7150/ijms.85704 38169594 PMC10758149

[B61] Lin Y. Lubman D. M. (2024). The role of N-glycosylation in cancer. Acta Pharm. Sin. B 14 (3), 1098–1110. 10.1016/j.apsb.2023.10.014 38486989 PMC10935144

[B62] Lin B. Qing X. Liao J. Zhuo K. (2020). Role of protein glycosylation in host-pathogen interaction. Cells 9 (4), 1022. 10.3390/cells9041022 32326128 PMC7226260

[B63] Liu L. Liu J. Shao D. Deng Q. Tang H. Liu Z. (2017). Comprehensive genomic profiling of lung cancer using a validated panel to explore therapeutic targets in East Asian patients. Cancer Sci. 108 (12), 2487–2494. 10.1111/cas.13410 28949084 PMC5715245

[B64] Liu Y. Chen X. Evan T. Esapa B. Chenoweth A. Cheung A. (2025). Folate receptor alpha for cancer therapy: an antibody and antibody-drug conjugate target coming of age. MAbs 17 (1), 2470309. 10.1080/19420862.2025.2470309 40045156 PMC11901361

[B65] Lu Y. F. Chang Y. H. Chen Y. J. Hsieh M. S. Lin M. W. Hsu H. H. (2024). Proteomic profiling of tumor microenvironment and prognosis risk prediction in stage I lung adenocarcinoma. Lung Cancer 191, 107791. 10.1016/j.lungcan.2024.107791 38621342

[B66] Lu J. Liu X. Ji X. Jiang Y. Zuo A. Guo Z. (2025). Predicting PD-L1 status in NSCLC patients using deep learning radiomics based on CT images. Sci. Rep. 15 (1), 12495. 10.1038/s41598-025-91575-y 40216830 PMC11992188

[B67] Mahajan A. Kania V. Agarwal U. Ashtekar R. Shukla S. Patil V. M. (2024). Deep-learning-based predictive imaging biomarker model for EGFR mutation status in non-small cell lung cancer from CT imaging. Cancers (Basel) 16 (6), 1130. 10.3390/cancers16061130 38539465 PMC10968632

[B68] Manfredi M. Brandi J. Di Carlo C. Vita Vanella V. Barberis E. Marengo E. (2019). Mining cancer biology through bioinformatic analysis of proteomic data. Expert Rev. Proteomics 16 (9), 733–747. 10.1080/14789450.2019.1654862 31398064

[B69] Medina V. A. Rivera E. S. (2010). Histamine receptors and cancer pharmacology. Br. J. Pharmacol. 161 (4), 755–767. 10.1111/j.1476-5381.2010.00961.x 20636392 PMC2992892

[B70] Mena F. Ienco D. Dantas C. F. Interdonato R. Dengel A. (2025). Multi-modal co-learning for Earth observation: enhancing single-modality models *via* modality collaboration. Mach. Learn. 114 (12), 279. 10.1007/s10994-025-06903-0

[B71] Messner C. B. Demichev V. Wang Z. Hartl J. Kustatscher G. Mülleder M. (2023). Mass spectrometry-based high-throughput proteomics and its role in biomedical studies and systems biology. Proteomics 23 (7-8), e2200013. 10.1002/pmic.202200013 36349817

[B72] Muro K. Feinstein T. Baranda J. Bonta I. Yanagitani N. Gersten T. (2025). Enfortumab vedotin in patients with advanced non-small cell lung cancer after disease progression on platinum- and PD-1/PD-L1 inhibitor-containing regimens: phase 2 international multicenter EV-202 study. Eur. J. Cancer 227, 115603. 10.1016/j.ejca.2025.115603 40819431

[B73] Naseer I. Akram S. Masood T. Rashid M. Jaffar A. (2023). Lung cancer classification using modified U-Net based lobe segmentation and nodule detection. IEEE Access 11, 60279–60291. 10.1109/ACCESS.2023.3285821

[B74] Nikanjam M. Pérez-Granado J. Gramling M. Larvol B. Kurzrock R. (2025). Nectin-4 expression patterns and therapeutics in oncology. Cancer Lett. 622, 217681. 10.1016/j.canlet.2025.217681 40209851 PMC12132789

[B75] O'Shannessy D. J. Yu G. Smale R. Fu Y. S. Singhal S. Thiel R. P. (2012). Folate receptor alpha expression in lung cancer: diagnostic and prognostic significance. Oncotarget 3 (4), 414–425. 10.18632/oncotarget.519 22547449 PMC3380576

[B76] Ovbude S. T. Sharmeen S. Kyei I. Olupathage H. Jones J. Bell R. J. (2024). Applications of chromatographic methods in metabolomics: a review. J. Chromatogr. B Anal. Technol. Biomed. Life Sci. 1239, 124124. 10.1016/j.jchromb.2024.124124 38640794 PMC11618781

[B77] Pantel K. Speicher M. R. (2016). The biology of circulating tumor cells. Oncogene 35 (10), 1216–1224. 10.1038/onc.2015.192 26050619

[B78] Pei Q. Luo Y. Chen Y. Li J. Xie D. Ye T. (2022). Artificial intelligence in clinical applications for lung cancer: diagnosis, treatment and prognosis. Clin. Chem. Lab. Med. 60 (12), 1974–1983. 10.1515/cclm-2022-0291 35771735

[B79] Ren F. Fei Q. Qiu K. Zhang Y. Zhang H. Sun L. (2024). Liquid biopsy techniques and lung cancer: diagnosis, monitoring and evaluation. J. Exp. Clin. Cancer Res. 43 (1), 96. 10.1186/s13046-024-03026-7 38561776 PMC10985944

[B80] Sahu A. Nema P. Rajak D. Purohit A. Rawal R. Soni V. (2025). Exploring AI tools and multi-omics for precision medicine in lung cancer therapy. Cytokine Growth Factor Rev. 84, 135–157. 10.1016/j.cytogfr.2025.06.001 40544104

[B81] Shang X. Zhang C. Kong R. Zhao C. Wang H. (2024). Construction of a diagnostic model for small cell lung cancer combining metabolomics and integrated machine learning. Oncologist 29 (3), e392–e401. 10.1093/oncolo/oyad261 37706531 PMC10911920

[B82] Shimada Y. Kudo Y. Maehara S. Amemiya R. Masuno R. Park J. (2022). Radiomics with artificial intelligence for the prediction of early recurrence in patients with clinical stage IA lung cancer. Ann. Surg. Oncol. 29 (13), 8185–8193. 10.1245/s10434-022-12516-x 36070112

[B83] Shur J. D. Doran S. J. Kumar S. Ap Dafydd D. Downey K. O'Connor J. P. B. (2021). Radiomics in oncology: a practical guide. Radiographics 41 (6), 1717–1732. 10.1148/rg.2021210037 34597235 PMC8501897

[B84] Song K. J. Choi S. Kim K. Hwang H. S. Chang E. Park J. S. (2024). Proteogenomic analysis reveals non-small cell lung cancer subtypes predicting chromosome instability, and tumor microenvironment. Nat. Commun. 15 (1), 10164. 10.1038/s41467-024-54434-4 39580524 PMC11585665

[B85] Subramanian I. Verma S. Kumar S. Jere A. Anamika K. (2020). Multi-omics data integration, interpretation, and its application. Bioinform Biol. Insights 14, 1177932219899051. 10.1177/1177932219899051 32076369 PMC7003173

[B86] Sung H. Ferlay J. Siegel R. L. Laversanne M. Soerjomataram I. Jemal A. (2021). Global cancer statistics 2020: GLOBOCAN estimates of incidence and mortality worldwide for 36 cancers in 185 countries. CA Cancer J. Clin. 71 (3), 209–249. 10.3322/caac.21660 33538338

[B87] Suwinski R. Giglok M. Galwas-Kliber K. Idasiak A. Jochymek B. Deja R. (2019). Blood serum proteins as biomarkers for prediction of survival, locoregional control and distant metastasis rate in radiotherapy and radio-chemotherapy for non-small cell lung cancer. BMC Cancer 19 (1), 427. 10.1186/s12885-019-5617-1 31068179 PMC6507220

[B88] Tamura N. Fujiwara Y. Hashimoto T. Shiraishi H. Kitano S. Shimizu T. (2020). Correlation between the expression of folate receptor alpha (FRα) and clinicopathological features in patients with lung adenocarcinoma. Lung Cancer 145, 152–157. 10.1016/j.lungcan.2020.05.002 32450493

[B89] Tao Y. Xing S. Zuo S. Bao P. Jin Y. Li Y. (2023). Cell-free multi-omics analysis reveals potential biomarkers in gastrointestinal cancer patients' blood. Cell Rep. Med. 4 (11), 101281. 10.1016/j.xcrm.2023.101281 37992683 PMC10694666

[B90] Telu K. H. Yan X. Wallace W. E. Stein S. E. Simón-Manso Y. (2016). Analysis of human plasma metabolites across different liquid chromatography/mass spectrometry platforms: cross-Platform transferable chemical signatures. Rapid Commun. Mass Spectrom. 30 (5), 581–593. 10.1002/rcm.7475 26842580 PMC5114847

[B91] Tong X. Zhao F. Thompson C. B. (2009). The molecular determinants of *de novo* nucleotide biosynthesis in cancer cells. Curr. Opin. Genet. Dev. 19 (1), 32–37. 10.1016/j.gde.2009.01.002 19201187 PMC2707261

[B92] Tyagi S. Talbar S. N. (2023). LCSCNet: a multi-level approach for lung cancer stage classification using 3D dense convolutional neural networks with concurrent squeeze-and-excitation module. Biomed. Signal Process. Control 80, 104391. 10.1016/j.bspc.2022.104391

[B93] Tyers M. Mann M. (2003). From genomics to proteomics. Nature 422 (6928), 193–197. 10.1038/nature01510 12634792

[B94] Valladares Ayerbes M. Aparicio Gallego G. Díaz Prado S. Jiménez Fonseca P. García Campelo R. Antón Aparicio L. M. (2008). Origin of renal cell carcinomas. Clin. Transl. Oncol. 10 (11), 697–712. 10.1007/s12094-008-0276-8 19015066

[B95] Vander Heiden M. G. Cantley L. C. Thompson C. B. (2009). Understanding the warburg effect: the metabolic requirements of cell proliferation. Science 324 (5930), 1029–1033. 10.1126/science.1160809 19460998 PMC2849637

[B96] Ventura D. Schindler P. Masthoff M. Görlich D. Dittmann M. Heindel W. (2023). Radiomics of tumor heterogeneity in (18)F-FDG-PET-CT for predicting response to immune checkpoint inhibition in therapy-naïve patients with advanced non-small-cell lung cancer. Cancers (Basel) 15 (8), 2297. 10.3390/cancers15082297 37190228 PMC10136892

[B97] Vinayanuwattikun C. Le Calvez-Kelm F. Abedi-Ardekani B. Zaridze D. Mukeria A. Voegele C. (2016). Elucidating genomic characteristics of lung cancer progression from *in situ* to invasive adenocarcinoma. Sci. Rep. 6, 31628. 10.1038/srep31628 27545006 PMC4992872

[B98] Wang S. Yu H. Gan Y. Wu Z. Li E. Li X. (2022). Mining whole-lung information by artificial intelligence for predicting EGFR genotype and targeted therapy response in lung cancer: a multicohort study. Lancet Digit. Health 4 (5), e309–e319. 10.1016/s2589-7500(22)00024-3 35341713

[B99] Wang X. Yu G. Yan Z. Wan L. Wang W. Cui L. (2023). Lung cancer subtype diagnosis by fusing image-genomics data and hybrid deep networks. IEEE/ACM Trans. Comput. Biol. Bioinform 20 (1), 512–523. 10.1109/tcbb.2021.3132292 34855599

[B100] Wang Z. Xie K. Zhu G. Ma C. Cheng C. Li Y. (2023). Early detection and stratification of lung cancer aided by a cost-effective assay targeting circulating tumor DNA (ctDNA) methylation. Respir. Res. 24 (1), 163. 10.1186/s12931-023-02449-8 37330511 PMC10276518

[B101] Wang F. Guo Z. Tang W. Cao W. Dong X. Xu Y. (2025). Lipidomic signatures as predictive biomarkers for early-onset lung cancer: identification and development of a risk prediction model. J. Adv. Res. 79, 679–690. 10.1016/j.jare.2025.03.045 40180245 PMC12766231

[B102] Wilkinson H. Saldova R. (2020). Current methods for the characterization of O-Glycans. J. Proteome Res. 19 (10), 3890–3905. 10.1021/acs.jproteome.0c00435 32893643

[B103] Witt C. (2011). European respiratory society/american thoracic society/international association for the study of lung cancer international multidisciplinary classification of lung adenocarcinoma: state of the art. J. Thorac. Oncol. 6 (8), 1451. 10.1097/JTO.0b013e318224643b 21847068

[B104] Wu Y. L. Zhou C. Liam C. K. Wu G. Liu X. Zhong Z. (2015). First-line erlotinib *versus* gemcitabine/cisplatin in patients with advanced EGFR mutation-positive non-small-cell lung cancer: analyses from the phase III, randomized, open-label, ENSURE study. Ann. Oncol. 26 (9), 1883–1889. 10.1093/annonc/mdv270 26105600

[B105] Yang T. Wang X. Jin Y. Yao X. Sun Z. Chen P. (2025). Deep learning radiopathomics predicts targeted therapy sensitivity in EGFR-mutant lung adenocarcinoma. J. Transl. Med. 23 (1), 482. 10.1186/s12967-025-06480-9 40301933 PMC12039126

[B106] Zeng W. Zheng S. Mao Y. Wang S. Zhong Y. Cao W. (2021). Elevated N-Glycosylation contributes to the cisplatin resistance of non-small cell lung cancer cells revealed by membrane proteomic and glycoproteomic analysis. Front. Pharmacol. 12, 805499. 10.3389/fphar.2021.805499 35002739 PMC8728018

[B107] Zhang G. J. Zhou Y. B. (2023). Artificial intelligence and machine learning in clinical medicine. N. Engl. J. Med. 388 (25), 2397–2398. 10.1056/NEJMc2305287 37342935

[B108] Zhang W. Hu X. Zhou W. Tam K. Y. (2018). Liquid chromatography-tandem mass spectrometry method revealed that lung cancer cells exhibited distinct metabolite profiles upon the treatment with different pyruvate dehydrogenase kinase inhibitors. J. Proteome Res. 17 (9), 3012–3021. 10.1021/acs.jproteome.8b00184 30028142

[B109] Zhang Y. Sun L. Lei C. Li W. Han J. Zhang J. (2022). A sweet warning: mucin-type O-Glycans in cancer. Cells 11 (22), 3666. 10.3390/cells11223666 36429094 PMC9688771

[B110] Zhang W. Zhao L. Zheng T. Fan L. Wang K. Li G. (2024). Comprehensive multi-omics integration uncovers mitochondrial gene signatures for prognosis and personalized therapy in lung adenocarcinoma. J. Transl. Med. 22 (1), 952. 10.1186/s12967-024-05754-y 39434164 PMC11492473

[B111] Zhang L. Wu Y. Li S. Guo M. Zhao J. Cao C. (2025). Proteomic analysis of extracellular vesicles identifies CDCP1 as critical metastasis-related glycoprotein in lung cancer. J. Extracell. Vesicles 14 (7), e70128. 10.1002/jev2.70128 40693605 PMC12281461

[B112] Zhao G. He F. Wu C. Li P. Li N. Deng J. (2018). Betaine in inflammation: mechanistic aspects and applications. Front. Immunol. 9, 1070. 10.3389/fimmu.2018.01070 29881379 PMC5976740

[B113] Zhao Y. Zhang D. Meng B. Zhang Y. Ma S. Zeng J. (2024). Integrated proteomic and glycoproteomic analysis reveals heterogeneity and molecular signatures of brain metastases from lung adenocarcinomas. Cancer Lett. 605, 217262. 10.1016/j.canlet.2024.217262 39341452

[B114] Zhou Y. Xu X. Song L. Wang C. Guo J. Yi Z. (2020). The application of artificial intelligence and radiomics in lung cancer. Precis. Clin. Med. 3 (3), 214–227. 10.1093/pcmedi/pbaa028 35694416 PMC8982538

[B115] Zhou Y. Guo Y. Ran M. Shan W. Granchi C. Giovannetti E. (2023). Combined inhibition of pyruvate dehydrogenase kinase 1 and lactate dehydrogenase a induces metabolic and signaling reprogramming and enhances lung adenocarcinoma cell killing. Cancer Lett. 577, 216425. 10.1016/j.canlet.2023.216425 37805163

[B116] Zhou J. Xu Y. Liu J. Feng L. Yu J. Chen D. (2024). Global burden of lung cancer in 2022 and projections to 2050: incidence and mortality estimates from GLOBOCAN. Cancer Epidemiol. 93, 102693. 10.1016/j.canep.2024.102693 39536404

[B117] Zhu Q. Fei L. (2025). Cross-ViT based benign and malignant classification of pulmonary nodules. PLoS One 20 (2), e0318670. 10.1371/journal.pone.0318670 39908279 PMC11798455

[B118] Zhu J. Ma J. Wang X. Ma T. Zhang S. Wang W. (2016). High expression of PHGDH predicts poor prognosis in non-small cell lung cancer. Transl. Oncol. 9 (6), 592–599. 10.1016/j.tranon.2016.08.003 27916294 PMC5143353

